# Prediction of survival after eribulin chemotherapy for breast cancer by absolute lymphocyte counts and progression types

**DOI:** 10.1186/s12957-021-02441-w

**Published:** 2021-11-15

**Authors:** Tamami Morisaki, Shinichiro Kashiwagi, Yuka Asano, Wataru Goto, Koji Takada, Sae Ishihara, Masatsune Shibutani, Hiroaki Tanaka, Kosei Hirakawa, Masaichi Ohira

**Affiliations:** 1 Department of Breast and Endocrine Surgery, Osaka City University Graduate School of Medicine, 1-4-3 Asahi-machi, Abeno-ku, Osaka, 545-8585 Japan; 2 Department of Gastrointestinal Surgery, Osaka City University Graduate School of Medicine, 1-4-3 Asahi-machi, Abeno-ku, Osaka, 545-8585 Japan

**Keywords:** Eribulin, Breast cancer, RECIST, Prognostic marker, Absolute lymphocyte counts

## Abstract

**Background:**

In the Response Evaluation Criteria for Solid Tumors (RECIST) diagnostic criteria, the concepts of progression by preexisting disease (PPL) and progression by new metastases (PNM) have been proposed to distinguish between the progression types of cancer refractory to treatment. According to the tumor biology of cancer progression forms, the “PPL” form indicates invasion, and the “PNM” form indicates metastasis. On the other hand, recent studies have focused on the clinical importance of inflammatory markers as indicators of the systemic tumor immune response. In particular, absolute lymphocyte count (ALC) is an indicator of the host’s immune response. Thus, we developed a new measure that combined progression form with ALC. In this study, we clinically validated the combined assessment of progression form and ALC in eribulin chemotherapy.

**Methods:**

From August 2011 to April 2019, a total of 486 patients with locally advanced or metastatic breast cancer (MBC) underwent treatment. In this study, only 88 patients who underwent chemotherapy using eribulin were included. The antitumor effect was evaluated based on the RECIST criteria, version 1.1. To measure ALC, peripheral blood samples collected before eribulin treatment were used. The cut-off value for ALC in this study was 1500/μl, based on previous studies.

**Results:**

The PPL group (71 patients, 80.7%) had significantly longer progression-free survival (PFS) (*p* = 0.022, log-rank) and overall survival (OS) (*p* < 0.001, log-rank) than the PNM group (17 patients, 19.3%). In the 51 patients with ALC < 1500/μl, the PPL group had a significantly better prognosis than the PNM group (PFS: *p* = 0.035, OS: *p* < 0.001, log-rank, respectively). On the other hand, in the 37 patients with ALC ≥ 1500/μl, the PPL group had a better OS compared with the PNM group (*p* = 0.055, log-rank), but there was no significant difference in PFS between the two groups (*p* = 0.541, log-rank). Furthermore, multivariate analysis that validated the effect of OS showed that high ORR and “high-ALC and PPL” were factors for a good prognosis (*p* < 0.001, *HR* = 0.321; *p* = 0.036, *HR* = 0.290).

**Conclusions:**

The progression form of PNM had a worse prognosis than PPL in patients treated with eribulin. In breast cancer patients with eribulin chemotherapy, good systemic immune status, such as ALC ≥ 1500/μl, was associated with less progression, particularly metastasis, and better prognosis. Furthermore, the biomarker “high-ALC (ALC ≥ 1500/μl) and PPL” was particularly useful as a prognostic marker following eribulin chemotherapy.

## Background

The Response Evaluation Criteria for Solid Tumors (RECIST) plays an important role in determining the response to chemotherapy for solid tumors as well as the treatment strategy [1]. In the RECIST diagnostic criteria, the concepts of progression by preexisting disease (PPL) and progression by new metastases (PNM) have been proposed to distinguish between the progression types of cancer refractory to treatment [2, 3]. Since both PPL and PNM are evaluated as “progression disease (PD)” by the RECIST diagnostic criteria, the difference in the form of progression has not influenced the choice of treatment. However, according to the tumor biology of cancer progression forms, the “PPL” form indicates invasion, and the “PNM” form indicates metastasis. Our previous study showed that patients with PPL who had good tumor immune microenvironment conditions had a good prognosis after eribulin chemotherapy [4].

On the other hand, recent studies have focused on the clinical importance of inflammatory markers as indicators of the systemic tumor immune response [5, 6]. The *in vivo* inflammatory response contributes to cancer progression. The peripheral blood neutrophil–lymphocyte ratio (NLR), lymphocyte–monocyte ratio (LMR), and platelet–lymphocyte ratio (PLR) of cancer patients have been proposed as indicators of the systemic inflammatory response [7–11]. In addition, several studies have reported that these factors predicted the prognosis of various carcinomas [12–15]. These inflammatory markers reflect a systemic tumor immune response. In particular, the absolute lymphocyte count (ALC) is an indicator of the host’s immune response.

In a phase III clinical trial on patients with locally advanced or metastatic breast cancer (MBC), eribulin significantly prolonged the overall survival (OS) (study 305, EMBRACE) [16]. Furthermore, the survival curve showed a characteristic pattern called the delayed separation curve in immunotherapy. Thus, this pattern may reflect the effects of eribulin on tumor immune response. A retrospective analysis of this trial showed that ALC was a useful marker in predicting the therapeutic effect of eribulin chemotherapy [17]. Additionally, real-world data on MBC patients treated with eribulin have reported on NLR, ALC prognosis, and predictors of therapeutic efficacy [18, 19].

We hypothesized that the combination of both the “form of PD” and the “host’s immune systemic marker, ALC” was a more sensitive indicator than ALC alone. Thus, we developed a new measure that combined progression form with ALC. In this study, we clinically validated the combined assessment of progression form and ALC in eribulin chemotherapy.

## Methods

### Patient background

**Fig. 1:**
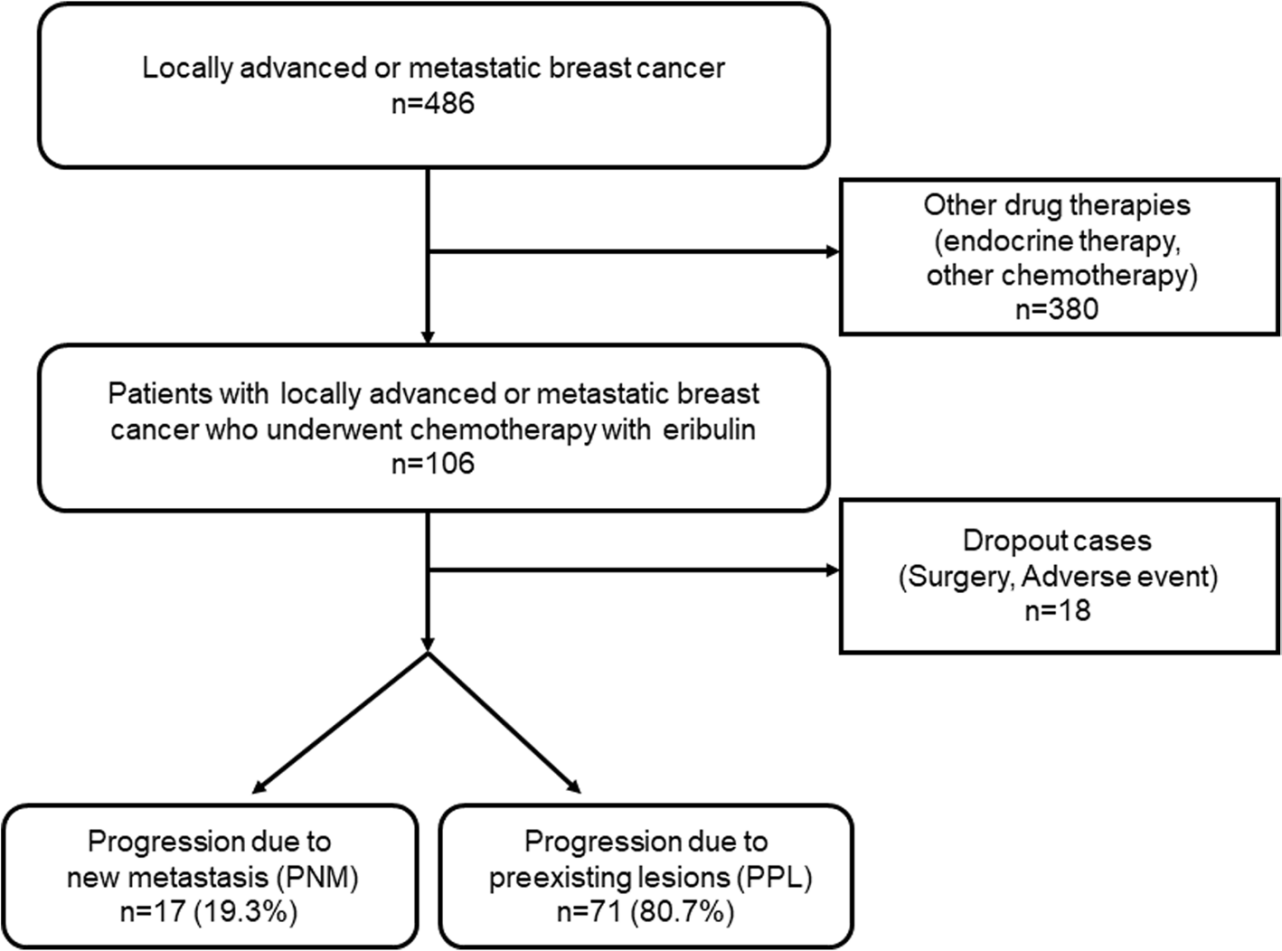
Consort diagram. From August 2011 to April 2019, a total of 486 patients with MBC underwent treatment at the Osaka City University Hospital. In this study, only 88 patients who underwent chemotherapy using eribulin were included, and 380 patients who were administered with other drug therapies, such as endocrine therapy or other chemotherapy regimens, and 18 patients who dropped out due to surgery or adverse events were excluded

From August 2011 to April 2019, a total of 486 patients with MBC underwent treatment at the Osaka City University Hospital. In this study, only 88 patients who underwent chemotherapy using eribulin were included, and 380 patients who were administered with other drug therapies, such as endocrine therapy or other chemotherapy regimens, and 18 patients who dropped out due to surgery or adverse events were excluded (Fig. 1). This dataset of eribulin chemotherapy was partially used in previous studies [20–22]. The median follow-up time for eribulin chemotherapy patients was 478 days (range: 50–2267 days). The chemotherapy regimen consisted of one course of treatment for 21 days (3 weeks), and eribulin mesylate (1.4 mg/m^2^) was intravenously administered on days 1 and 8 [16, 23]. This protocol was followed repeatedly until PD was evaluated or therapy was discontinued due to severe adverse events. Chemotherapy was administered in all cases on an outpatient basis.

Based on the efficacy of this regimen, the objective response rate (ORR), OS, and progression-free survival (PFS) were determined. ORR was evaluated by adding complete response (CR) and partial response (PR). OS was defined as the time from the date of treatment initiation to death (daily). PFS was defined as the time from the date of treatment initiation to the date of death or PD confirmation, whichever was earlier (daily). The antitumor effect was evaluated based on the RECIST criteria, version 1.1 [1].

### Classification based on progression types

According to the RECIST guidelines, PPL is described as an increase by at least 20% in the sum of the diameter of the lesion evaluated or an absolute increase of 5 mm or more in the sum of the diameter from the lowest sum of the diameter to date [2, 3]. PNM was defined as a new lesion, indicative of disease progression, identified during a follow-up session. If PPL and PNM were observed simultaneously during the evaluation, it was considered a PNM.

### Blood sample analysis

To measure ALC, peripheral blood samples collected before eribulin treatment were used. The percentage of white blood cells was measured using a Coulter LH750 blood analyzer (Beckman Coulter, Brea, CA, USA). The cut-off value for ALC in this study was 1500/μl, based on previous studies [17, 18, 24]. ALC values ≥ 1500 /μl were considered high, while values below 1500/μl were low.

### Statistical analysis

We used SPSS® Statistics version 25 statistical software (IBM, Armonk, NY, USA) for the statistical analysis. To analyze whether clinical parameters were associated with ALC, the chi-squared test or Fisher’s exact test was used as appropriate. The association with survival was analyzed using Kaplan–Meier plots and the log-rank test. Cox proportional hazards models were used to calculate univariate and multivariate hazard ratios (HRs) for the study parameters with 95% confidence intervals (CIs). The selection of variables in the multivariate analysis included a backward stepwise method. For all statistical tests, a *p* value of less than 0.05 was considered statistically significant.

### Ethics statement

This study complies with the provisions of the Declaration of Helsinki (64th WMA General Assembly, Fortaleza, Brazil, October 2013). This study consisted of a retrospective chart review. While receiving treatment, patients provided written informed consent for the use of patient data in later research studies. This research protocol was approved by the Ethics Committee of Osaka City University (#926).

## Results

### Differences in progression types and prognostic analysis

**Fig. 2:**
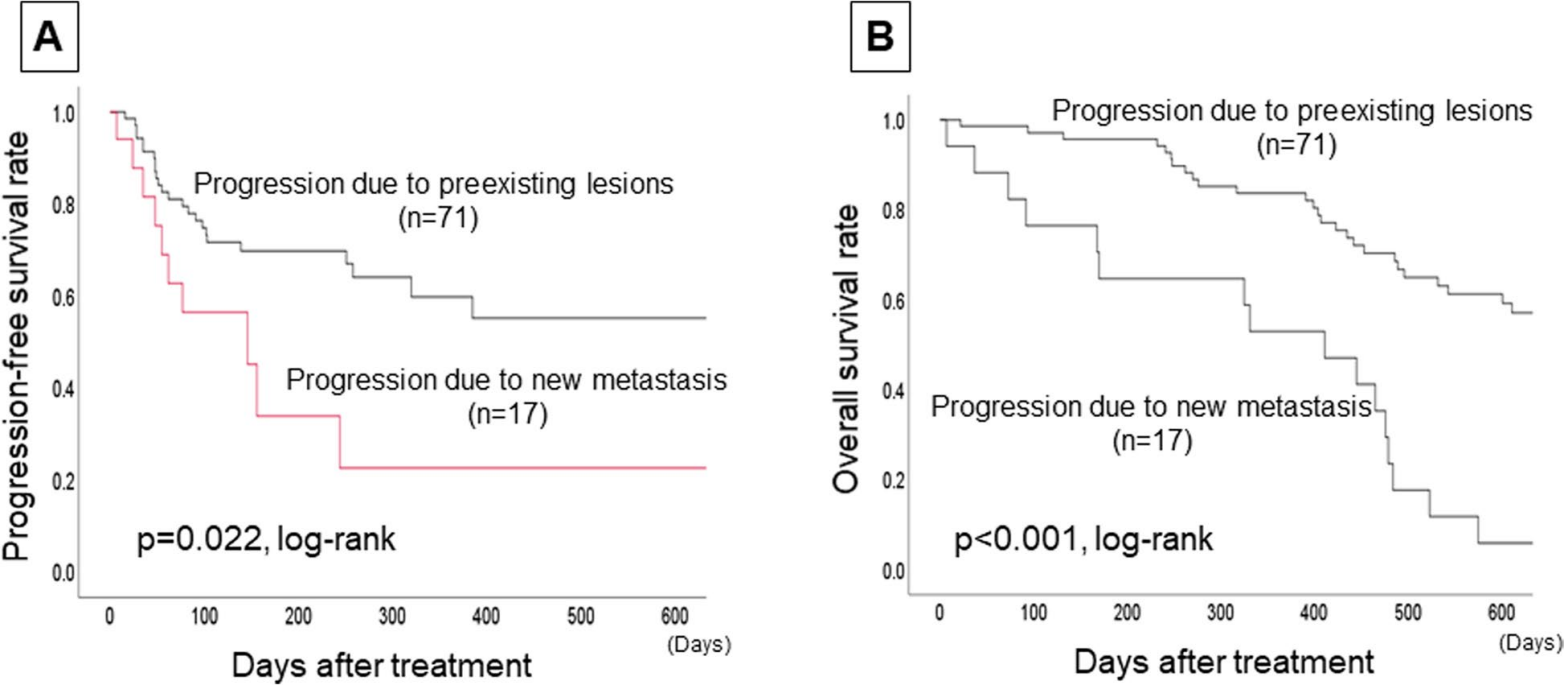
Differences in progression types and prognostic analysis. The 71 patients of the PPL group had significantly longer PFS (*p* = 0.022, log-rank) (**A**) and OS (*p* < 0.001, log-rank) (**B**) than the 17 patients of the PNM group

Among the 106 MBC patients who received chemotherapy with eribulin, 88 were included in the study, and 18 patients were excluded. Among them, 17 patients (19.3%) were PNM, and 71 patients (80.7%) were PPL. The PPL group had significantly longer PFS (*p* = 0.022, log-rank) and OS (*p* < 0.001, log-rank) than the PNM group (Fig. 2).

### Absolute lymphocyte counts and differences in progression types

Among the 88 patients, 37 (42.0%) were included in the high-ALC group, and 51 (58.0%) were in the low-ALC group. Among the 17 patients in the PNM group, 5 were classified in the high-ALC group (29.4%), and 12 were in the low-ALC group (70.6%). Among the 71 patients in the PPL group, 32 (45.1%) were classified in the high-ALC group, and 39 (54.9%) were in the low-ALC group. When the groups were divided based on the difference in ALC, there was no significant difference between the clinicopathological parameter and ALC (Table 1).

**Table 1:** Correlations between absolute lymphocyte counts and clinicopathological parameters in 88 patients with eribulin chemotherapy for locally advanced or metastatic breast cancer

Parameters	All breast cancer (*n* = 88)			Progression due to new metastasis (*n* = 17)			Progression due to preexisting lesions (*n* = 71)		
	High (*n* = 37)	Low (*n* = 51)	*p* value	High (*n* = 5)	Low (*n* = 12)	*p* value	High (*n* = 32)	Low (*n* = 39)	*p* value
Age at chemotherapy									
≤ 63	24 (64.9%)	28 (54.9%)		3 (60.0%)	6 (50.0%)		21 (65.6%)	22 (56.4)	
> 63	13 (35.1%)	23 (45.1%)	0.348	2 (40.0%)	6 (50.0%)	0.563	11 (34.4%)	17 (43.6%)	0.293
Degree of progress									
Locally advanced	11 (29.7%)	13 (25.5%)		1 (20.0%)	5 (41.7%)		10 (31.3%)	8 (20.5%)	
Visceral metastases	26 (70.3%)	38 (74.5%)	0.659	4 (80.0%)	7 (58.3%)	0.395	22 (68.7%)	31 (79.5%)	0.301
HR (ER and/or PgR) status									
Negative	12 (32.4%)	21 (41.2%)		1 (20.0%)	6 (50.0%)		11 (34.4%)	15 (38.5%)	
Positive	25 (67.6%)	30 (58.8%)	0.403	4 (80.0%)	6 (50.0%)	0.278	21 (65.6%)	24 (61.5%)	0.722
HER2 status									
Negative	33 (89.2%)	47 (92.2%)		5 (100.0%)	12 (100.0%)		28 (87.5%)	35 (89.7%)	
Positive	4 (10.8%)	4 (7.8%)	0.633	0 (0.0%)	0 (0.0%)	-	4 (12.5%)	4 (10.3%)	0.527
Ki67									
Low	21 (56.8%)	30 (58.8%)		2 (40.0%)	4 (33.3%)		19 (59.4%)	26 (66.7%)	
High	16 (43.2%)	21 (41.2%)	0.846	3 (60.0%)	8 (66.7%)	0.605	13 (40.6%)	13 (33.3%)	0.526
Nuclear grade									
1, 2	24 (64.9%)	38 (74.5%)		3 (60.0%)	8 (66.7%)		21 (65.6%)	30 (76.9%)	
3	13 (35.1%)	13 (25.5%)	0.328	2 (40.0%)	4 (33.3%)	0.605	11 (34.4%)	9 (23.1%)	0.292
Objective response rate									
ORR	23 (62.2%)	18 (35.3%)		3 (60.0%)	2 (16.7%)		20 (62.5%)	16 (41.0%)	
Non-ORR	14 (37.8%)	33 (64.7%)	0.013	2 (40.0%)	10 (83.3%)	0.117	12 (37.5%)	23 (59.0%)	0.072

*HR* hormone receptor, *ER* estrogen receptor, *PgR* progesterone receptor, *HER2* human epidermal growth factor receptor, *ORR* objective response rate

### Effects of ALC and differences in progression type upon prognosis

**Fig. 3:**
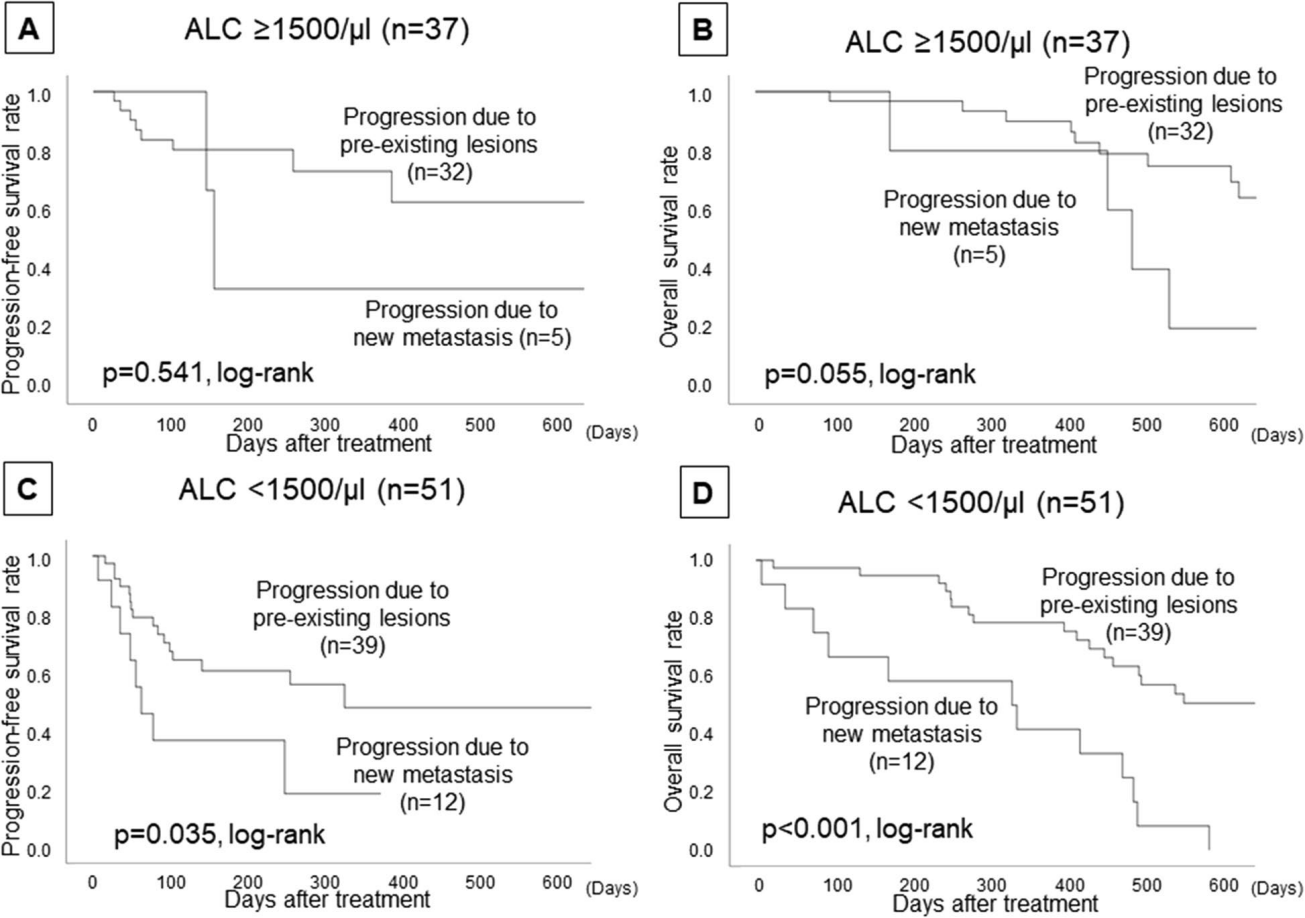
Effects of ALC and differences in progression type upon prognosis. In the 37 patients with ALC ≥ 1500/μl, the PPL group had a better OS compared with the PNM group (**B**: *p* = 0.055, log-rank), but there was no significant difference in PFS between the two groups (**A**: *p* = 0.541, log-rank). On the other hand, in the 51 patients with ALC < 1500/μl, the PPL group had a significantly better prognosis than the PNM group (**C**: PFS *p* = 0.035, **D**: OS *p* < 0.001, log-rank, respectively)

In the 51 patients with ALC < 1500/μl, the PPL group had a significantly better prognosis than the PNM group (PFS: *p* = 0.035, OS: *p* < 0.001, log-rank, respectively) (Fig. 3). On the other hand, in the 37 patients with ALC ≥ 1500/μl, the PPL group had a better OS compared with the PNM group (*p* = 0.055, log-rank), but there was no significant difference in PFS between the two groups (*p* = 0.541, log-rank).

**Fig. 4:**
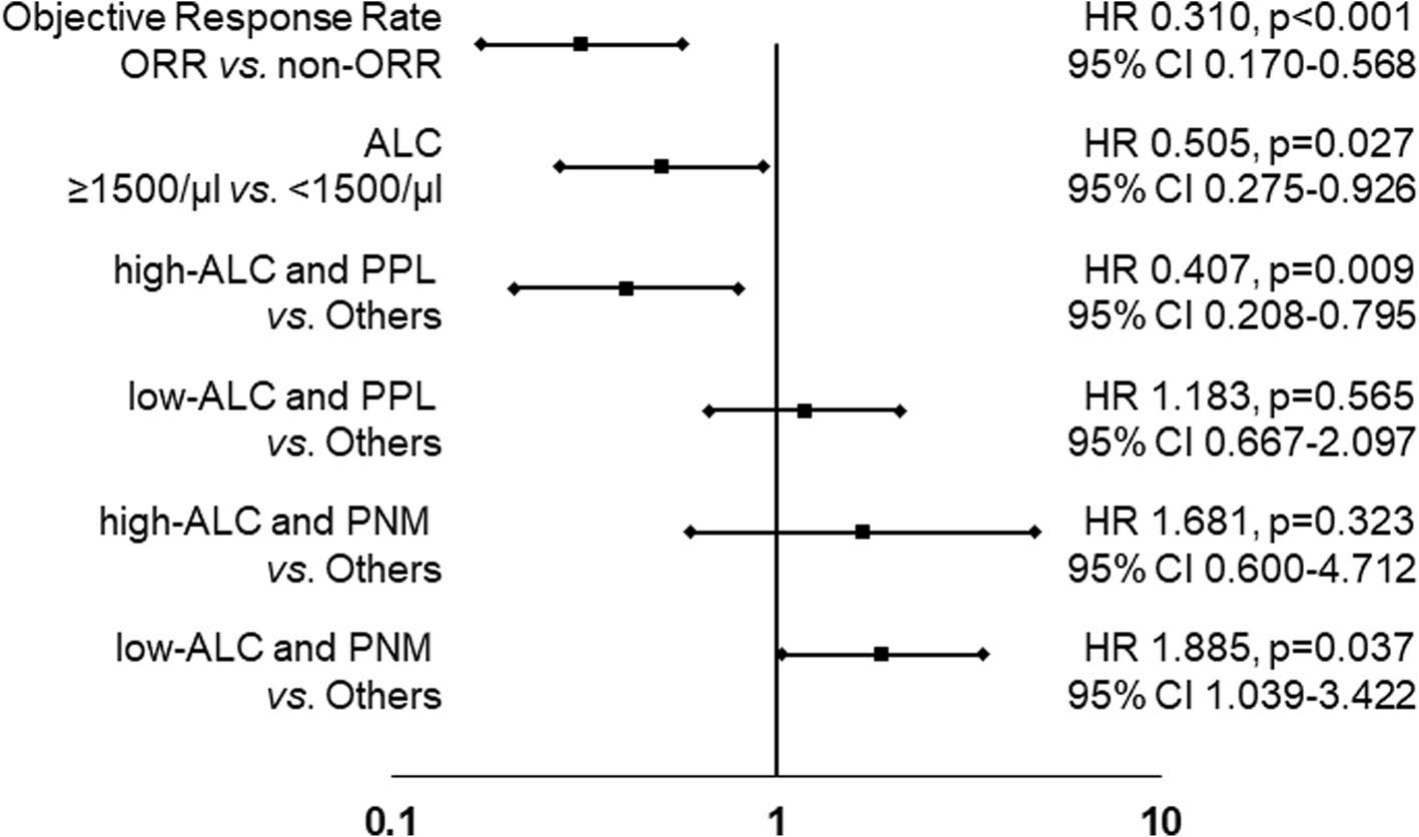
Forest plots. A univariate analysis that validated the effect of OS showed that high ORR, high-ALC, and “high-ALC and PPL” were factors for a good prognosis (*p* < 0.001, *HR* = 0.310, 95% CI: 0.170–0.568; *p* = 0.027, *HR* = 0.505, 95% CI: 0.275–0.926; *p* = 0.009, *HR* = 0.407, 95% CI: 0.208–0.795)

**Fig. 5:**
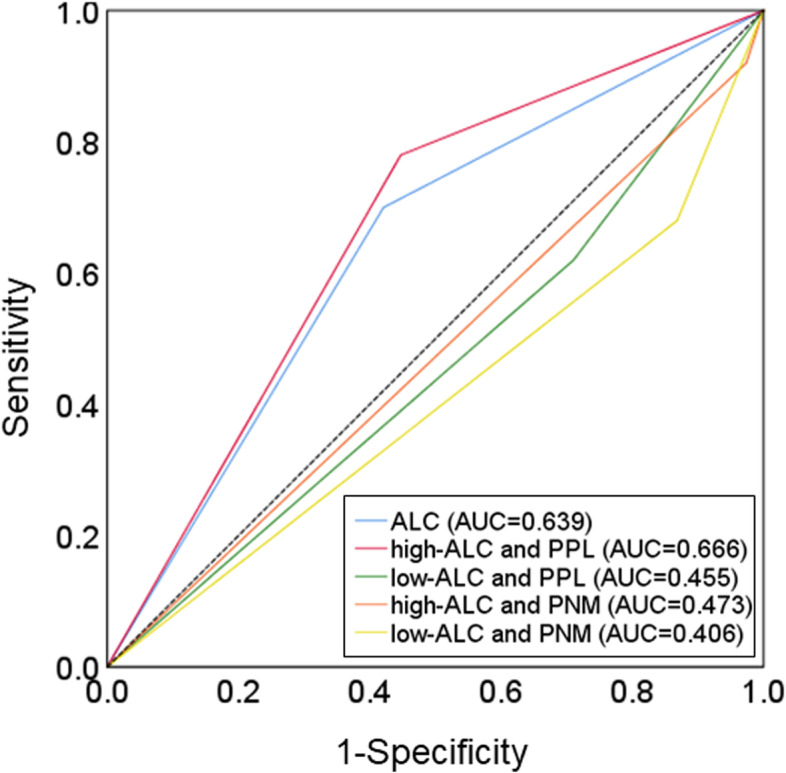
Receiver operating characteristic (ROC) analysis. ROC analysis showed that the results for “high-ALC and PPL” (area under the curve (AUC): 0.666) were better than those for the ALC (AUC: 0.639), “low-ALC and PPL” (AUC: 0.455), “high-ALC and PNM” (AUC: 0.473), and “low-ALC and PNM” (AUC: 0.406)

A univariate analysis that validated the effect of OS showed that high ORR, high-ALC, and “high-ALC and PPL” were factors for a good prognosis (*p* < 0.001, *HR* = 0.310, 95% CI: 0.170–0.568) (*p* = 0.027, *HR* = 0.505, 95% CI: 0.275–0.926) (*p* = 0.009, *HR* = 0.407, 95% CI: 0.208–0.795) (Fig. 4). Receiver operating characteristic (ROC) analysis showed that the results for “high-ALC and PPL” (area under the curve (AUC): 0.666) were better than those for the ALC (AUC: 0.639), “low-ALC and PPL” (AUC: 0.455), “high-ALC and PNM” (AUC: 0.473), and “low-ALC and PNM” (AUC: 0.406) (Fig. 5). Furthermore, multivariate analysis demonstrated that ORR was the strongest independent factor for a favorable prognosis (*p* < 0.001, *HR* = 0.321, 95% CI: 0.171–0.602). In addition, “high-ALC and PPL” was another independent factor for a favorable prognosis (*p* = 0.036, *HR* = 0.290, 95% CI: 0.091–0.923) (Table 2).

**Table 2:** Univariate and multivariate analysis with respect to overall survival in 88 patients with eribulin chemotherapy for locally advanced or metastatic breast cancer

		Univariate analysis			Multivariate analysis		
Parameters		Hazard ratio	95% CI	*p* value	Hazard ratio	95% CI	*p* value
Age at chemotherapy	≤ 63 *vs*. > 63	0.653	0.366–1.164	0.148			
Degree of progress	Locally advanced *vs*. visceral metastases	0.622	0.328–1.178	0.144			
HR (ER and/or PgR)	Positive *vs*. negative	0.630	0.359–1.107	0.108			
HER2	Positive *vs*. negative	0.366	0.089–1.506	0.164			
Ki67	≤ 14% *vs*. > 14%	1.401	0.803–2.446	0.235			
Nuclear grade	1, 2 *vs*. 3	1.501	0.842–2.673	0.168			
Objective response rate	ORR *vs.* non-ORR	0.310	0.170–0.568	< 0.001	0.321	0.171–0.602	< 0.001
ALCs	≥ 1500/μl *vs*. < 1500/μl	0.505	0.275–0.926	0.027	0.424	0.138–1.300	0.133
Progression	Progression due to preexisting lesions and high-ALCs *vs*. others	0.407	0.208–0.795	0.009	0.290	0.091–0.923	0.036
Progression	Progression due to preexisting lesions and low-ALCs *vs*. others	1.183	0.667–2.097	0.565			
Progression	Progression due to new metastasis and high-ALCs *vs*. others	1.681	0.600–4.712	0.323			
Progression	Progression due to new metastasis and low-ALCs *vs*. others	1.885	1.039–3.422	0.037	1.654	0.846–3.234	0.142

*HR* hormone receptor, *ER* estrogen receptor, *PgR* progesterone receptor, *HER2* human epidermal growth factor receptor, *ORR* objective response rate, *ALC* absolute lymphocyte count, *CI* confidence interval

## Discussion

Eribulin chemotherapy for MBC patients has been shown to prolong OS in international phase III clinical trials (Study 305, EMBRACE) [16]. Prolonging OS in MBC, which is biologically mild and has more treatment options, is difficult. Bevacizumab combination therapy has a high response rate and has been shown to improve PFS, but it did not significantly affect OS (E2100, AVADO, RIBBON-1) [25–28]. Meanwhile, eribulin chemotherapy benefitted MBC patients by improving the OS. However, there was no significant difference in PFS, and the reason for this is being investigated [16]. The modulating effect of eribulin on the tumor microenvironment through tumor vascular remodeling and epithelial–mesenchymal transition (EMT) suppression is a possible mechanism for OS prolongation [29–31]. In our previous study, which analyzed tissue specimens collected after eribulin treatment, tumor microenvironment (TME) improvement, such as reduced tumor hypoxia and EMT suppression, was observed in the responders [32]. Furthermore, a study using the same tissue specimens showed decreased expression of programmed cell death protein (PD)-1, programmed death ligand-1 (PD-L1), and forkhead box P3 (FOXP3) as well as increased expression of CD8 [33]. The eribulin-resistant MDA-MB-231 breast cancer cell line also showed lower CD274 (PD-L1) expression than the parental cell line [34]. These results indicated an improvement in tumor immunity with eribulin chemotherapy.

ALC and NLR, which are indicators of systemic tumor immune response, have been reported to be prognostic and predictive of therapeutic response in patients treated with eribulin. This was supported by the results of the EMBRACE study [17–19, 24]. We have also shown that local and systemic tumor immune responses are linked via transforming growth factor-β (TGF-β) [20].

The RECIST diagnostic criteria for PD were divided into PPL and PNM. Those with PNM had a worse prognosis than those with PPL in Studies 305 and 301 [3]. In the present study, the progression form of PNM had a worse prognosis than PPL in patients treated with eribulin. PNM is associated with peripheral tissue invasion and metastasis to other organs, explaining the poor prognostic course. On the other hand, PPL is associated with peripheral tissue invasion only, without metastasis [2–4, 35]. In other words, differences in progression patterns are related to dynamic changes in the TME. The rates of PNM in this study were 13.5% (5/37) in high-ALC cases (ALC ≥ 1500/μl) and less than 23.5% (12/51) in low-ALC cases (ALC < 1500/μl). That is, patients with higher ALC had a lesser form of PNM progression. Although ALC is a useful biomarker for eribulin chemotherapy, its mechanism has not been validated until now. The results of our study suggest that a good systemic immune status contributes to benefit in terms of OS. A good local tumor immune microenvironment reduces the progression of PNM. Our previous study showed that systemic and local tumor immune responses were linked to eribulin chemotherapy [20], and the present study showed that the form of progression was a factor. In other words, high-ALC cases (ALC ≥ 1500/μl) had more PPL and better prognosis than the progression form of PNM due to their better immune status. Furthermore, the use of the combination of “form of PD” and “host’s immune systemic marker, ALC” was more sensitive than using ALC alone. In particular, “high ALC and PPL” were independent favorable prognostic factors for overall survival. Good systemic immune status and progressive forms of PPL suggested a good prognosis. However, indicators such as “low ALC and PPL”, “high ALC and PNM”, and “low ALC and PNM” were not useful as biomarkers.

This study had limitations since it involved a retrospective cohort analysis with a small sample size. However, this is the first report to capture the mechanism behind the role of ALC as a useful biomarker in eribulin chemotherapy in a progressive form of cancer. We developed a new biomarker, “high-ALC and PPL”, which was found to be more sensitive than ALC alone. In the future, these biomarkers should also be considered in clinical practice to determine the best treatment options.

## Conclusions

The progression form of PNM had a worse prognosis than PPL in patients treated with eribulin. In breast cancer patients with eribulin chemotherapy, good systemic immune status, such as ALC ≥ 1500/μl, was associated with less progression, particularly metastasis, and better prognosis. Furthermore, the biomarker “high-ALC (ALC ≥ 1500/μl) and PPL” was particularly useful as a prognostic marker following eribulin chemotherapy.

## Data Availability

The datasets used and/or analyzed during the current study are available from the corresponding author on reasonable request.

## Abbreviations

ALC: Absolute lymphocyte count
AUC: Area under the curve
CI: Confidence interval
CR: Complete response
EMT: Epithelial–mesenchymal transition
ER: Estrogen receptor
H.E.: Hematoxylin and eosin
FOXP3: Forkhead box P3
HER2: Human epidermal growth factor receptor 2
HR: Hazard ratio
LMR: Lymphocyte–monocyte ratio
MBC: Locally advanced or metastatic breast cancer
NLR: Neutrophil–lymphocyte ratio
ORR: Objective response rate
OS: Overall survival
PD: Progression disease
RECIST: Response Evaluation Criteria for Solid Tumors
ROC: Receiver operating characteristic
PD-L1: Programmed death ligand-1
PFS: Progression-free survival
PgR: Progesterone receptor
PLR: Platelet–lymphocyte ratio
PNM: Progression by new metastases
PPL: Progression by preexisting disease
PR: Partial response
TME: Tumor microenvironment
TGF-β: Transforming growth factor-β
